# Quantifying the Multidimensionality of Abstract Concepts: An Italian Database

**DOI:** 10.3390/brainsci15030222

**Published:** 2025-02-21

**Authors:** Virginia Maria Borsa, Maria Arioli, Riccardo Verni, Nicola Canessa, Stefano F. Cappa, Eleonora Catricalà

**Affiliations:** 1 IUSS Cognitive Neuroscience (ICoN) Center, Scuola Universitaria Superiore IUSS, 27100 Pavia, Italy; virginia.borsa@iusspavia.it (V.M.B.); nicola.canessa@iusspavia.it (N.C.); stefano.cappa@iusspavia.it (S.F.C.); 2 Department of Human and Social Sciences, University of Bergamo, 24129 Bergamo, Italy; maria.arioli@unibg.it; 3 Cognitive Neuroscience Laboratory of Pavia, Istituti Clinici Scientifici Maugeri IRCCS, 27100 Pavia, Italy

**Keywords:** exclusivity, abstract concepts, dimension rating

## Abstract

**Background:** The embodied cognition approach, as applied to concrete knowledge, is centred on the role of the perceptual and motor aspects of experience. To extend the embodied framework to abstract knowledge, some studies have suggested that further dimensions, such as affective or social experiences, are relevant for the semantic representations of abstract concepts. The objective of this study is to develop a measure that can quantitatively capture the multidimensional nature of abstract concepts. **Methods**: We used dimension-rating methods, known to be suitable, to account for the semantic representations of abstract concepts, to develop a new database of 964 Italian words, rated by 542 participants. Besides classical psycholinguistic variables (i.e., concreteness, imageability, familiarity, age of acquisition, semantic diversity) and affective norms (i.e., valence, arousal), we collected ratings on selected dimensions characterizing the semantic representations of abstract concepts, i.e., introspective, mental state, quantitative, spatial, social, moral, theoretical, and economic dimensions. The measure of exclusivity was incorporated to quantify the number of dimensions, and the respective relevance, for each concept. Concepts with a high value of exclusivity rely on only one/a few dimension/s with high value on the respective rating scale. **Results**: A multidimensional representation characterized most abstract concepts, with two robust major clusters. The first was characterized by dense intersections among introspective, mental state, social, and moral dimensions; the second, less interconnected, cluster revolved around quantitative, spatial, theoretical, and economic dimensions. Quantitative, theoretical, and economic concepts obtained higher exclusivity values. **Conclusions**: The present study contributes to the investigation of the semantic organization of abstract words and supports a controlled selection and definition of stimuli for clinical and research settings.

## 1. Introduction

Investigations of the structure of the semantic memory system, and of its neurobiological underpinnings, have largely focused on concrete concepts. Many neurobiological models within the embodied framework suggest that the distinct brain regions involved in the processing of sensory and motor information are differentially involved in conceptual representations, with each dimension being differently relevant in characterizing specific classes of concepts (e.g., visual knowledge for animals, motor knowledge for tools) [1].

Supporting this view, concepts with a high relevance of acoustic features (e.g., telephone) engage brain systems for sounds perception (i.e., left posterior and middle temporal gyri) [2]; those with a strong relevance of colour features (e.g., taxi) elicit activations in brain structures involved in colour perception, mainly located in the fusiform gyrus [3]; and concepts associated with odour-related properties (e.g., garlic) involve the olfactory cortex [4].

Drawing on this extensive literature, in recent years, abstract concepts have also received evidence in favour of their multimodal representation [5,6,7]. Starting with Barsalou’s proposal on the relevance of introspection, in recent years, several aspects of experience have been proposed to support abstract semantic representations [5,6,8,9,10,11]. Behavioural studies, involving property listing and dimension rating, reported that modality-specific features (both sensory and motor), alongside introspective, affective, social, and magnitudinal aspects, as well as verbal associations, are relevant in the representation of abstract words [7,9,12,13,14,15,16,17,18,19]. Accordingly, the organization of abstract concepts may reflect different dimensions characterizing distinct subtypes of abstract words, possibly grounded in brain regions coding for the corresponding experience [5,11,20,21].

A consistent pattern of results provided by clinical, neuroimaging, and neurophysiological studies supported the existence of different types of abstract concepts, associated with partially non-overlapping neural correlates; see [5] for a review.

Convergent results by neuroimaging and neurophysiological studies with healthy subjects [22,23,24,25,26,27,28,29,30], as well as clinical studies on patients diagnosed with the semantic variant of primary progressive aphasia [31,32] suggest that the neural representation of social concepts involves the anterior temporal lobe, and particularly its superior portion, previously associated with functions pertaining to the domain of social cognition [33,34]. Quantity-related concepts mostly involve the intraparietal sulcus [30] known to be associated with numerical processing [35]. In line with this finding, a selective impairment for quantity-related concepts has been reported in patients with the cortico-basal syndrome, in which parietal areas are involved by neurodegeneration [32].

It is unlikely, however, that a unique dimension can capture the entire meaning of an abstract concept, independently of its relevance. Mental state concepts, for example, involve areas related to mentalizing but also regions involved in automatic speech and lip movements [21]. As in the case of concrete concepts [36,37], and possibly even more due to their fuzzy inter-categorical demarcations and their context-dependent meaning, many abstract concepts are expected to engage a multidimensional representation, reflected by a widespread involvement of several brain areas, contributing to more than one dimension [11,37].

A comprehensive neural model is currently hard to accomplish, but the need to simultaneously also consider additional experiential brain systems, such as those responsible for temporal, spatial and affective dimensions, is clear in order to also account for the neural representation of abstract concepts [5,12,18,19,20,21,38,39]. The dynamic properties of this representation system are shown, for instance, by the increase in activation in brain areas associated with social–emotional scene observation during the processing of words corresponding to scientific psychological concepts after academic training [39].

Dimension rating, in which participants are asked to rate the importance/relevance of specific dimensions—such as introspection, quantity, and social relevance—for the meaning of a given word, has been considered a useful behavioural approach to characterize the representation of abstract concepts [5,12,14,18,19,38]. Only a few studies adopting this approach are available, with considerable variation in the number of dimensions (ranging from 1 [14] to 65 [12]) or words (ranging from 400 [18] to 17,940 [40]) considered. Measuring only one dimension, such as the social aspect of abstract concepts [14], may lead to the underestimation of potential co-occurring inherent dimensions of social conceptual processing, e.g., those related to emotional and/or mental states, according to the typical multidimensionality of abstract knowledge [5]. With a few exceptions [20,21], studies assessing a specific class of abstract concepts selected stimuli with a high value on the dimension under investigation, controlling for psycholinguistic variables but neglecting the possible contribution of other dimensions (see, for example, [23,25] for social concepts or [41,42] for emotional words). Consider, for example, the two words *meeting* and *crime*, both potentially “social” but with the moral dimension playing a key role only for the latter. It is, thus, clear that a high value on a specific dimension does not exclude the occurrence of high values on other dimensions.

Nine databases are available for the Italian language: four of them collected ratings for traditional psycholinguistic measures and affective norms [43,44,45,46]; four collected ratings on variables mainly related to *sensorimotor* experience [38,47,48,49], while the last and most recent one is mainly focused on aspects related to ecological/technological dimensions [50]. Semantic dimensions, i.e., experience-related properties characterizing conceptual representation and possibly grounded in specific neural correlates [12,18,19], have only seldom been assessed in Italian databases. The semantic dimensions that are most closely linked to abstract concepts are under-represented and scattered across the different databases and include affective [45,49,50], introspective [49,50], social [38,50], mental states [38,50], and scientificity [50].

The present study aims to provide a more comprehensive consideration of the conceptual representation of abstract concepts, based on several known experiences contributing to their meaning (see [12,19]), while being aware that this cannot be exhaustive. In addition to the traditional psycholinguistic variables, the resulting representation contains entries corresponding to introspection, mental state, emotional valence, and arousal, alongside social, moral, theoretical, and economic dimensions, as well as quantity and space.

A key aim of this study is to develop a measure that can quantitatively capture the multidimensional nature of abstract concepts, based on the results from dimension-rating studies. For this purpose, we adapted the *exclusivity* measure [48,49,51,52], which was originally used to assess concrete knowledge related to sensorimotor features. In the present context, we aimed to determine the position of each word along a unidimensional-to-multidimensional semantic continuum, using the eight semantic dimensions in the dataset (i.e., social, theoretical, economic, spatial, quantitative, mental state, moral, introspective). This metric provides insights into the degree of multidimensionality of each concept, with words that receive similar ratings across multiple dimensions being associated with low exclusivity scores.

## 2. Methods

### 2.1. Participants

A total of 542 healthy young participants (363 females; age, mean: 24.89 ± 5.22; years of education, mean: 16.89 ± 2.62) volunteered for this study. The study protocol was approved by the local ethical committee and implemented in accordance with the principles of the Declaration of Helsinki.

### 2.2. Stimuli and Dimensions

Nine hundred and sixty-four words were rated for 14 psycholinguistic variables and semantic dimensions.

#### 2.2.1. Words

We selected 964 Italian nouns, i.e., 157 denoting well-known classical concrete categories, namely including 66 animals, 62 tools, 19 fruits, and 10 words without a specific category label (e.g., kiss), and 807 words denoting 8 different a priori categories, namely 122 positive and negative emotions (e.g., happiness, sadness); 65 mental states (e.g., hallucination); 117 quantitative (e.g., abundance) and spatial (e.g., perimeter) concepts; 144 positive, negative, and neutral social (e.g., friendship, discrimination, authority); 127 positive and negative moral (e.g., honesty, greediness) concepts; 69 theoretical (e.g., adverb) and 135 positive, negative, and neutral economic (e.g., gain, loss, budget) concepts; and 10 denoting generic abstract concepts (e.g., destiny). These a priori categories were based on agreement between two authors (in case of disagreement, a further author was asked for a classification). Importantly, this a priori classification was only used to select a large number of stimuli trying to possible cover all dimensions of interest and not to identify and encapsulate stimuli in a rigid label, which was not used in subsequent analyses.

Importantly, the distinction between abstract and concrete nouns falls outside the aim of the present study, which is rather focused on the characterization—in terms of dimensions and exclusivity—of words not corresponding to the classical well-studied concrete categories such as tools or animals. The latter categories were, thus, used only as fillers, to ensure the presence of both extremes in scales such as imageability and to obtain data that might be compared with other databases assessing the same measures both on abstract and concrete words (e.g., [44]). The statistical analyses were based only on data for abstract words.

#### 2.2.2. Psycholinguistic Variables and Semantic Dimensions

All the words were rated for concreteness (CNC), imageability (IMG), familiarity (FAM), and age of acquisition (AoA), with instructions taken from Della Rosa et al. [44], on 7-point Likert scales in which 1 indicated *highly abstract*, *difficult to image*, and *very unfamiliar* in CNC, IMG, and FAM, respectively. In addition, participants indicated the age at which they learnt the word (AoA), by estimating the approximate age at which they were able to understand the meaning of the word even if they did not use it themselves and/or were not able to read and/or write it.

The semantic diversity scale (SEM_DIV) was used to identify the number of contexts with which a word could be associated and its different meanings [53,54], with scores ranging from 1 (single context) to 7 (multiple contexts).

Emotional valence and arousal were assessed on a 9-point Likert scale based on the Self-Assessment Manikin [55].

Then, we adopted six semantic dimensions based on previous works [12,18,19], as well as two novel dimensions related to theoretical [56] and economic concepts. Overall, the stimuli were rated according to the following dimensions:

- Introspection (INTRO), referring to subjective and internal experiences, like emotions, self-referential thoughts, and mental activity [57,58].
- Mental states (MENT_ST), referring to beliefs, desires, intentions related to the self and others, related to Theory of Mind [12,18].
- Quantity (QUANT), referring to size, amount, capacity, etc. [18].
- Space (SPACE), referring to place, area, directions, etc. [18].
- Social aspects (SOC), referring to how much a word is linked to a social situation or to an interaction among people, both in terms of inclusion and exclusion [12,18].
- Moral aspects (MOR), referring to morality, rules, and anything that govern human behaviour [18].
- Theoretical aspects (THEOR), referring to theoretical disciplines and technical languages like “*equation*” or “*bigram*” [56].
- Economic aspects (ECO), referring to economy, finance, and trading operations.

These dimensions were rated on a 7-point Likert scale, ranging from “Not at all” related to (1), through “Neutral” (4), to “Strongly” related to each variable (7). The participants were instructed to rate stimuli carefully by using the entire scale.

### 2.3. Procedure

The 964 included words were randomly divided into 24 lists: 20 lists composed of 40 words and 4 lists composed of 41 words. Two different versions of each list were created to randomize the order of the rated dimensions while keeping the same set of words.

Each of the 24 lists was rated on 7/8 dimensions. Following previous related studies [44], emotional valence and arousal were always presented together, therefore resulting in 8 dimensions per list. Ultimately, every list was rated for all 15 dimensions through an online survey (SoSci; version 2.5.00-i1142). Every participant was presented with two different lists, each including 40 or 41 words, for a total of 80 or 81 assessed words. Therefore, each participant rated all the dimensions across the two lists but for distinct words. This procedure ensured that each word was rated for all dimensions by at least 20 participants (range = 20–27). The participants could stop, and then resume, the rating procedure at any time.

### 2.4. Data Trimming

Two experimenters performed a visual inspection of each participant’s responses to exclude the following:

- Participants who failed to follow instructions or to complete the ratings or provided random answers;
- Participants who used the same value of the Likert scale for more than 85% of responses for all ratings.

These criteria led to the exclusion of 23 participants. Subsequently, for each dimension and item, we examined all the responses, to exclude responses greater than 3 standard deviations from the item mean. For each dimension, the percentage of excluded responses was lower than 2% (from 0.17% to 1.76%).

### 2.5. Data Analyses

Data analysis was performed using IBM SPSS Statistics (version 21) software.

### 2.6. Reliability

To account for inter-rater reliability, Cronbach’s alpha [59] was computed for each dimension. Values above 0.6 were considered acceptable [60].

### 2.7. Concreteness and Imageability of Abstract Words

We first compared the CNC and IMG means across the classical concrete categories of concepts (*n* = 157) and those of our interest (*n* = 807). We then quantified the percentage of the 807 words of interest with a value of CNC included in the range of the 157 classical concrete concepts.

#### 2.7.1. Exclusivity (E)

The subsequent analysis focused only on abstract words (*n* = 807).

Following [51], exclusivity (E) was defined in the case of concrete words as the extent to which a particular concept is perceived through a single modality) (range (MIN-MAX)/sum of ratings across modalities × 100). In keeping with our aim to extend the measure of exclusivity to abstract concepts, we calculated the extent to which each concept is considered to be experienced through a unique dimension. We computed E by taking into account the eight semantic dimensions potentially characterizing the meaning of abstract concepts, i.e., introspective, mental states, quantitative, spatial, social, moral, theoretical, and economic dimensions. Following Lynott and Connell [51], the E score was calculated for each word as the range of values among the 8 selected dimensions divided by the sum of values of each dimension (range (MIN-MAX)/sum of ratings across dimensions × 100). An entirely multimodal concept would be scored equally on all dimensions, resulting in the lowest exclusivity score, whilst the most exclusive concept would be scored high only on a unique dimension. Note that exclusivity was not calculated for concrete concepts, as their relevant features, such as vision or motor ones, were not considered in this study. The distributions of E were than divided into three levels, i.e., low, medium, and high, according to a score lower than the 25th percentile (<15.24), ranging between the 25th and 75th (≥15.24 ≤ 20.29) percentile, or higher than the 75th percentile (>20.29), respectively.

Correlation analyses were then performed for all abstract words between the following:

- All psycholinguistic variables and semantic dimensions;
- Exclusivity and SEM_DIV, as a measure of validity.

#### 2.7.2. Identifying the Most Representative Abstract Words for Each Dimension

To identify words mainly associated with particular kinds of experiences, we searched for words obtaining a score higher than 3.5 on one or more of the 8 dimensions of interest. These words were thus labelled as theoretical, and/or social, and/or spatial, and/or introspective, and/or mental state, and/or moral, and/or economic, and/or quantitative on the basis of the dimension/s scored over 3.5. Note that a single word could be representative for more than one dimension, i.e., when it obtained a value >3.5 on at least two dimensions (i.e., *comitiva* was labelled as social and quantitative, as it was rated more than 3.5 for both socialness and quantitative dimensions). Accordingly, a high value on a specific dimension does not imply that a concept cannot have high values on other dimensions. The number of words in each intersection among dimensions, i.e., the number of words sharing the contribution of different dimensions, in the different combinations, was calculated and visualized using the “UpSset plots”, for all representative words and for words with high, intermediate, and low E levels, separately (http://www.ehbio.com/test/venn/#/, accessed on 3 October 2024; [61]).

Finally, we used a Venn network to provide a visual structure highlighting the interconnectedness among the 8 dimensions of interest. The Venn network goes beyond the traditional limits of standard Venn diagrams, surpassing simple representations of intersections and unions. It skilfully depicts relationships within sets by assigning each set as a parent node and linking individual elements to their corresponding parent nodes through edges.

The Venn networks were developed with the BarnesHut solver and provided, for all representative words and separately for each E level, to show the relationships among the 8 dimensions. Each dimension was designed as a parent node, and each individual word was connected to their respective parent nodes via edges [61]. This procedure allowed to show which dimensions contribute to characterize the same words and/or which dimensions uniquely underpin the representation for specific words. Near and distant dimensions will, thus, reflect a simultaneous contribution to many vs. few/no words.

## 3. Results

A new Italian dataset was created, including 964 concepts rated for 15 variables (see https://osf.io/6xyun/?view_only=062f49f57f9f4a6f8c73aab672c86dd2, accessed on 12 August 2023).

### 3.1. Reliability

Reliability measures were acceptable for all the dimensions: AoA (α = 0.92), FAM (α = 0.86), IMG (α = 0.94), CNC (α = 0.95), SEM_DIV (α = 0.87), mental state (α = 0.96), introspective (α = 0.96), moral (α = 0.92), economic (α = 0.96), quantitative (α = 0.92), spatial (α = 0.89), social (α = 0.93), theoretical (α= 0.82), valence (α = 0.96), and arousal (α = 0.90).

### 3.2. Concreteness and Imageability of Abstract Words

The CNC and IMG means of the 157 words denoting the classical concrete categories (mean = 6.88, range = 4.57–7; mean = 6.87, range = 4.20–7; respectively) were significantly different from those of the 807 words of interest (mean = 3.02; range = 1.11–6.95; mean = 3.21, range = 1.18–7; respectively) (*p* < 0.0001 in both cases). For 117 abstract concepts, concreteness values were included in the range of the 157 classical concrete concepts (see the Table 1). The range 4.57–7 was split in two parts, including values from 4.57 to 5.99, and from 6 to 7, respectively. A value > 6 at the CNC scale was observed for 98% and 17.95% of concrete and abstract concepts, respectively.

### 3.3. Exclusivity

**Figure 1:**
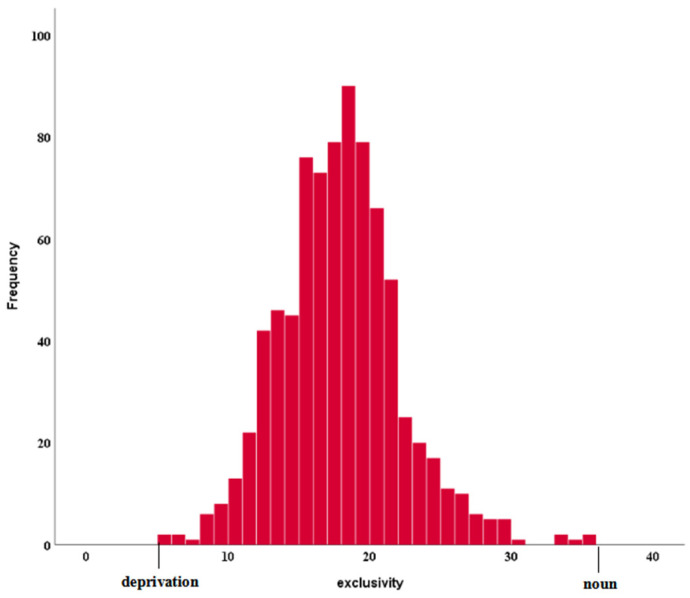
Distribution of exclusivity scores, with lowest (“deprivation”) and highest (“noun”) values on the left and right, respectively.

The exclusivity scores for the 807 abstract words ranged from 5.52 to 35.76 (see Figure 1). The means and standard deviations of exclusivity for the total sample of words and for the low, intermediate, and high E levels are displayed in Table 2. The word with the highest value of E, i.e., *noun*, obtained a value of more than 3.5 only on the theoretical dimension. Conversely, the word with the lowest E value, i.e., *deprivation*, obtained more than 3.5 on all semantic dimensions except space.

The correlations between all the psycholinguistic variables and the semantic dimensions for the whole set of abstract concepts (*n* = 807) are reported in Supplementary Table S1.

**Figure 2:**
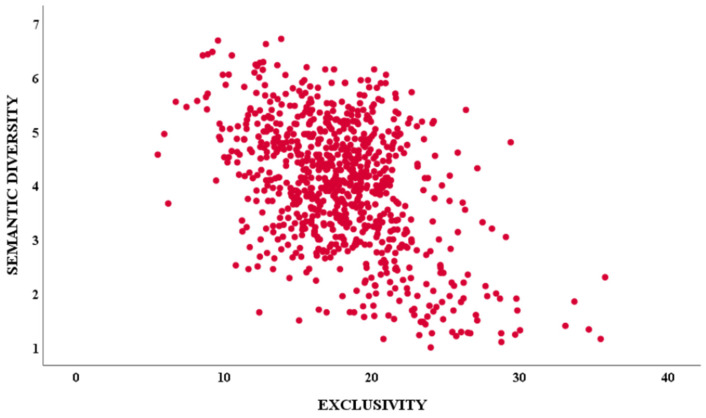
Scatterplot showing the correlation between exclusivity and semantic diversity on 807 abstract words. SEM DIV = semantic diversity.

A negative correlation was found between E and SEM_DIV (r = −0.47; *p* < 0.0001), suggesting that being associated with multiple contexts is related with a semantic representation characterized by a greater number of relevant dimensions (see Figure 2).

### 3.4. The Most Representative Abstract Words of Each Dimension

**Figure 3:**
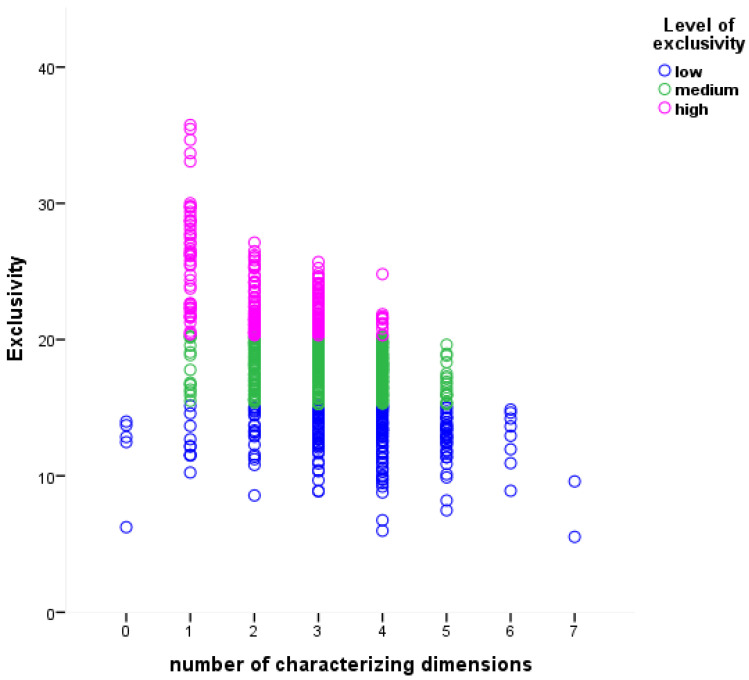
Graphical representation of the 807 words as divided in the three level of exclusivity (low in blue, medium in green, and high in pink) in terms of exclusivity value and number of characterizing dimensions (i.e., those with values > 3.5). Number of characterizing dimensions = 0 indicates words (*n* = 5) not relevant for any dimension (see text for details).

Overall, 802 words were representative for at least one dimension, while five concepts (0.62% of the total number of words) did not reach a score >3.5 in any dimension. Importantly, several concepts were considered representative of different dimensions, based on the number of dimensions on which they obtained a score above of 3.5 (see Figure 3). One dimension characterized only 9.1% of the representative words. However, the value of E does not depend only on the number of dimensions characterizing each concept (see Figure 3). While the two words *noun* and *style* were characterized by only one dimension, they did not have the same level of exclusivity. For instance, *noun* had a mean value of 6.63 on the theoretical scale and E = 35.76, while *style* had a value of 4.55 on the social scale and E = 16.84. Indeed, E captures the combination of the number of dimensions and their relevance in characterizing the word meaning.

The number of the most representative concepts varied across dimensions. In particular, we identified 272 theoretical, 233 economic, 245 quantitative, 135 spatial, 417 introspective, 420 mental state, 462 social, and 321 moral concepts. The means and standard deviations of exclusivity for each group of the most representative concepts (e.g., introspective, economic, social) are reported in Supplementary Table S2. The means and standard deviations of psycholinguistic variables and semantic dimensions for each level of exclusivity are reported in Supplementary Tables S3 and S4.

**Figure 4:**
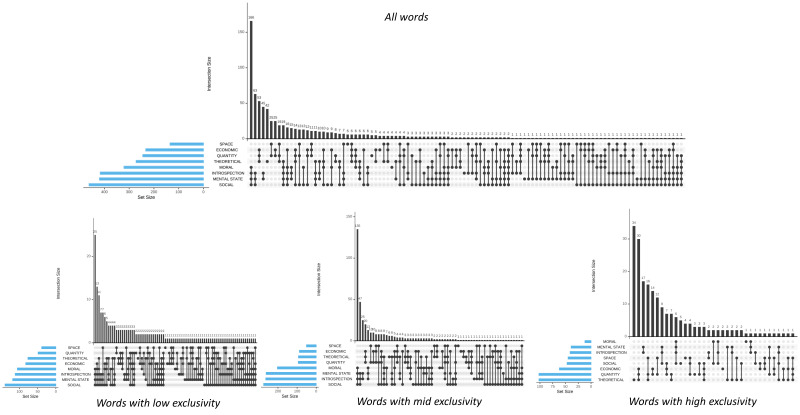
UpSet plots reporting the number of words sharing the same combinations among the 8 dimensions for the total of 802 representative words, as well as for words with low, mid, and high exclusivity. For each plot, the blue horizontal bar depicts the total words included not exclusively in each dimension; the vertical bar plot indicates the number of words in the corresponding combination of dimensions, which are in turn delineated by the matrix of connected dots. Individual points refer to words belonged to unique dimension, and connecting points represent words sharing a combination of dimensions, i.e., with two connected points denoting words sharing two dimensions, with three connected points denoting words sharing three dimensions, etc. For example, considering the first plot, the first column represents 166 words all represented by the combination of 4 dimensions (the 4 interconnected points), namely, moral, introspection, mental state, and social.

The UpSet plots in Figure 4 report the number of representative words for each dimension. Note that, in this case, each word may also be included in other dimensions, namely having a value >3.5 on one or more dimensions. In addition, the plots interestingly indicate the number of words exclusively characterized by only one dimension (when a point corresponding to a specific dimension is displayed, e.g., *n* = 46 for the theoretical dimension) or by a specific combination of dimensions (e.g., *n* = 166 for moral, introspection, mental state, and social dimensions).

**Figure 5:**
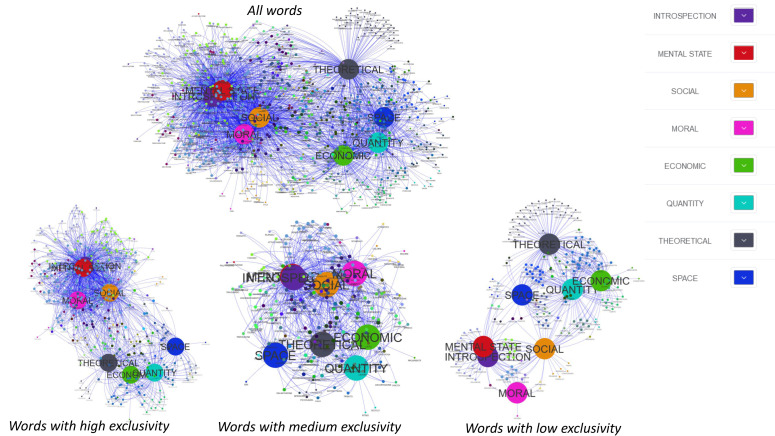
Venn network diagrams for the 8 dimensions, considering all the representative words (above), as well as words with low (bottom left), medium (bottom centred), and high (bottom right) levels of exclusivity; see text for details.

Network diagrams unveil the intricate relationships among the eight dimensions (see Figure 5). In particular, considering all the representative words, many intersections emerged among all the dimensions, although it was possible to differentiate two major clusters characterized by a large number of internal intersections. The first cluster included words represented by introspection, mental state, social, and moral dimensions, all sharing the highest number of non-empty intersections, namely 21% (see Figure 5). The second cluster, involving economic, spatial, quantitative, and theoretical dimensions, entails a smaller number of intersections highlighting a more heterogeneous structure and a greater separability among the dimensions.

A great number of intersections and overlap among dimensions is evident when low-exclusivity words are considered separately: in particular, considering introspection and mental state. Conversely, a high exclusivity level allows to highlight and distinguish concepts based on the most peculiar dimensions, with the exception of introspection and mental states that are still largely similar, as their contribution involved almost the same words. Theoretical, quantitative, and economic concepts were the most represented ones at the highest exclusivity level.

## 4. Discussion

The available evidence on the neural representation of conceptual knowledge indicates that concrete concepts are processed by distributed multimodal brain regions [2,3,4,37,62]. A multidimensional representation is likely involved also in the case of abstract concepts, as, in most cases, a single dimension cannot capture the entire meaning referring to the multiple facets of emotional–social–situational experiences [5,9,11,12,20,21].

To ease the quantification of multidimensionality and evaluate the relevance of each semantic dimension (i.e., emotions, mental states, social, moral, economic) in a sample of 807 abstract words, we adapted the measure of exclusivity previously adopted on object properties [49,51].

In the present work, the measure of exclusivity combines the number of dimensions with the value on the respective dimension-rating scale, with items that scored high on fewer dimensions being the most exclusive. Higher scores in semantic diversity were associated with lower exclusivity, showing the validity of the measure. Accordingly, from an embodied perspective [57,58], emotion and other abstract concepts such as “convince” are endowed with a situated conceptualization closely depending on the context in which an individual has experienced an emotion or a mental state. Abstract concepts, however, may be related to mental events occurring in different contexts. For example, we may experience fear in a situation of physical danger (e.g., walking across the woods in the middle of the night) as well as in a social situation (e.g., public speaking). Different contexts may, therefore, prompt specific relevant dimensions, thereby recruiting selective neural systems in charge of processing information relevant to the specific situation [63].

Theoretical concepts were found to be very peculiar with respect to the other classes of abstract concepts, as they were at the same time highly abstract in nature and very low in multidimensionality. This result fits with previous studies suggesting that theoretical words are mainly linguistic constructs, based to a very small extent on sensory information of any kind [13,38,56]. A complete multidimensional pattern was, in contrast, found for moral concepts, indicating that the moral dimension is intrinsically multifaceted.

The present study reported considerable heterogeneity in terms of exclusivity in the realm of abstract knowledge. Many intersections among the eight dimensions and with different combinations among them were reported, i.e., with 91% of words grounding on more than one dimension.

Moving from the highest through the intermediate to the lowest exclusivity levels leads to a gradual shift to increasingly multidimensional representations. In particular, the lowest exclusivity level reflects in many classes of concepts overlapping along several dimensions, with a coarse division in two major clusters. An almost complete overlap was in particular reported for introspection and mental states (contributing together to the meaning of the same words), which in turn were also similar to the moral and social dimensions. On the other hand, quantitative, spatial, economic, and theoretical dimensions also contributed to the meaning of the same words but to a smaller extent and with more heterogeneous combinations. Concepts with the highest exclusivity values were characterized by a highly relevant core dimension, in terms of value on the respective dimension-rating scale. In particular quantitative, spatial, theoretical, and economic concepts appeared to be less intersected than the other dimensions.

The overlap across dimensions for different types of concepts fits with the previous evidence. For example, the similarity between space and quantity has been already reported and clustered in a magnitude factor [18,19]. For example, spatial concepts like “*extension*” may capture quantity in terms of graduality.

The strong similarities among introspective, mental, social, and moral concepts suggest the presence of a “latent” factor, highlighting information that arises from the Self [19] and its respective metacognitive processes [64,65]. In addition, these similarities suggest that each dimension intrinsically captures multiple facets of the inner experience [7,8,14,38,64]. In particular, the overlapping representations of introspective and mental state concepts at all levels of exclusivity suggest that individuals tend to consider all introspective experiences as mind related, therefore associating inner experience with metacognitive processes [8,38,64] rather than with interoceptive experiences [66].

A recent review highlights considerable heterogeneity in the neural correlates of different types of abstract words that do not appear to converge on the same regions across studies [5]. For instance, in the case of mental states, eight different regions emerged from four studies, with over 50% of the studies showing non-converging results. According to our database, words representing mental states are generally associated with at least one other relevant dimension in 99.8% of cases, as shown in Figure 4. Among the 419 words in which mental states were a representative dimension, 51% also involved three other relevant dimensions, 25% involved two other dimensions, 19% involved one additional dimension, and 10% involved four more dimensions. For social words, fifteen different regions were identified across nine studies, with convergence occurring only in the superior anterior temporal region [5].

In addition to possible methodological issues, such a heterogeneous neural topography may reflect the multidimensionality of abstract concepts, which was not considered in previous studies, and the variability in the stimuli classified under specific categories of abstract concepts. Indeed, scoring high on a specific semantic dimension does not entail low scores on all the other ones, and—with few exceptions (see, for example, [20,21])—concepts have been usually assessed only for the dimension of interest, while neglecting other possible relevant dimensions. This was the case, for example, in studies focusing on social [23,25], emotional [41,42,67], or mental state [68,69] words, which in our studies were found to be multidimensional. Indeed, a concept like *honour* was classified as social by some authors (e.g., [23]) and as emotional by others (e.g., [67]). Similarly, concepts like *belief* or *desire* were classified as related to mental states by some authors [68,69] and to emotion by others [42,67]. According to our norms, these concepts belong to the medium-exclusivity range, in which a great overlap has been reported for introspection, mental states, moral, and social concepts, i.e., those represented by high values on all these dimensions.

### Limits

This study is limited by the lack of comparisons with concrete concepts and of norms for sensory and motor dimensions. However, we chose to focus on selected experiential dimensions for a restricted set of concepts due to practical considerations about the length of the experimental session. Future studies should cover a more comprehensive characterization of abstract concepts to better investigate their organization. In addition, the present results might be biased by the unbalanced gender distribution, with women representing 67% of our sample. However, the evidence provided by previous databases highlights either the lack of this information [12,16,17,19,44,51] or considerable heterogeneity in gender distribution, with female percentages at 73% [15], 68% [18], 65% [13], 63% [38], 46% [49], or 40% [14]. Accordingly, the effect of a gender is still to be investigated by future studies collecting the features and dimensions of abstract words.

## 5. Conclusions

In conclusion, the present findings show that high values on a relevant dimension are not necessarily and uniquely representative of a specific category of concepts, given the multidimensionality of the experience/semantic knowledge related to the majority of abstract concepts. Even if some dimensions are able to capture specific exclusive features of abstract knowledge, our results confirm that a quantitative multidimensional approach fits better with the representation of abstract concepts and should be taken into proper consideration when investigating their respective neural correlates. By reporting the high quantitative score for the multidimensionality of abstract words, alongside their class-specific differences, the present findings pave the way for future studies addressing the neuro-cognitive organization of semantic memory.

## Figures and Tables

**Table 1:** Number of abstract and concrete words included in the range of CNC values of the 157 concrete words, split into two parts.

CNC Range	*n* Concrete Concepts	*n* Abstract Concepts
4.57–5.99	3	96
6–7.00	154	21
Total	157	117

**Table 2:** Mean, standard deviation, and range of exclusivity, for the total number of words and for the three levels of exclusivity (low, medium, and high).

Exclusivity	Number of Words	Mean	Standard Deviation	Range
Total	807	17.9	4.22	5.52–35.76
Low	202	12.8	1.94	5.52–15.24
Medium	404	17.8	1.43	15.27–20.29
High	201	23.24	2.99	20.30–35.76

## Data Availability

Data or materials for the experiments are available at https://osf.io/6xyun/?view_only=062f49f57f9f4a6f8c73aab672c86dd2, accessed on 12 August 2023 and none of the experiments was preregistered.

## Supplementary Materials

The following supporting information can be downloaded at: https://www.mdpi.com/article/10.3390/brainsci15030222/s1, Table S1: Correlations between all psycholinguistic variables and all semantic dimensions, considering all abstract concepts (N = 807); Table S2: Mean and standard deviation of exclusivity total scores and for each level of exclusivity. Table S3: Mean and standard deviation of all psycholinguistic variables for all types of concepts, for each level of exclusivity (low, medium, and high). Table S4: Mean and standard deviation of all semantic dimensions for all types of concepts, for each level of exclusivity (low, medium, and high).
