# Supplementary material for: Quantifying the Multidimensionality of Abstract Concepts: An Italian Database
Source: Brain Sci. 2025 Feb 21;15(3):222. doi: 10.3390/brainsci15030222 (PMC11940458; doi:10.3390/brainsci15030222)
Supplement: Supplementary file 1 [file brainsci-15-00222-s001.zip › brainsci-3383855-supplementary.pdf]

Table S1: correlations between all psycholinguistic variables and all semantic dimensions, considering all abstract concepts (N=807).

		INTRO	MENT ST	SOC	MOR	ECO	THEOR	SPACE	QUANT	CNC	IMG	FAM	AoA	VAL	AROUSAL	SEM DIV
**INTRO**	r (Pearson)	1														
	Sign.															
	N	807														
**MENT ST**	r (Pearson)	,946**	1													
	Sign.	0,000														
	N	807	807													
**SOC**	r (Pearson)	,431**	,424**	1												
	Sign.	0,000	0,000													
	N	807	807	807												
**MOR**	r (Pearson)	,552**	,544**	,655**	1											
	Sign.	0,000	0,000	0,000												
	N	807	807	807	807											
**ECO**	r (Pearson)	-,508**	-,509**	-,109**	-,152**	1										
	Sign.	0,000	0,000	0,002	0,000											
	N	807	807	807	807	807										
**THEOR**	r (Pearson)	-,432**	-,405**	-,400**	-,298**	,183**	1									
	Sign.	0,000	0,000	0,000	0,000	0,000										
	N	806	806	806	806	806	806									
**SPACE**	r (Pearson)	-,391**	-,389**	-,301**	-,461**	0,011	0,065	1								
	Sign.	0,000	0,000	0,000	0,000	0,758	0,063									
	N	807	807	807	807	807	806	807								
**QUANT**	r (Pearson)	-,608**	-,615**	-,389**	-,507**	,577**	,191**	,523**	1							
	Sign.	0,000	0,000	0,000	0,000	0,000	0,000	0,000								
	N	807	807	807	807	807	806	807	807							
**CNC**	r (Pearson)	-,697**	-,707**	-,210**	-,408**	,405**	-0,013	,350**	,504**	1						
	Sign.	0,000	0,000	0,000	0,000	0,000	0,715	0,000	0,000							
	N	807	807	807	807	807	806	807	807	807						
**IMG**	r (Pearson)	-,490**	-,496**	-,079*	-,319**	,195**	-,239**	,320**	,348**	,806**	1					
	Sign.	0,000	0,000	0,025	0,000	0,000	0,000	0,000	0,000	0,000						
	N	807	807	807	807	807	806	807	807	807	807					
**FAM**	r (Pearson)	,251**	,219**	,235**	,104**	-,118**	-,440**	0,003	-0,037	0,068	,253**	1				
	Sign.	0,000	0,000	0,000	0,003	0,001	0,000	0,929	0,298	0,054	0,000					

	N	807	807	807	807	807	806	807	807	807	807	807				
**AoA**	r (Pearson)	-,135**	-,114**	-,124**	-0,031	,224**	,519**	-,184**	-0,019	-,134**	-,356**	-,729**	1			
	Sign.	0,000	0,001	0,000	0,380	0,000	0,000	0,000	0,587	0,000	0,000	0,000				
	N	807	807	807	807	807	806	807	807	807	807	807	807			
**VAL**	r (Pearson)	-0,006	0,023	-0,004	,144**	0,010	-0,010	-,094**	-,087*	0,001	-0,033	-,265**	,198**	1		
	Sign.	0,863	0,516	0,900	0,000	0,786	0,767	0,008	0,013	0,969	0,356	0,000	0,000			
	N	807	807	807	807	807	806	807	807	807	807	807	807	807		
**AROUSAL**	r (Pearson)	-,198**	-,223**	-,221**	-,267**	0,039	,252**	,188**	,189**	0,047	0,009	,087*	-,076*	-,781**	1	
	Sign.	0,000	0,000	0,000	0,000	0,272	0,000	0,000	0,000	0,186	0,796	0,013	0,032	0,000		
	N	807	807	807	807	807	806	807	807	807	807	807	807	807	807	
**SEM DIV**	r (Pearson)	,333**	,329**	,209**	,142**	-,162**	-,352**	,290**	0,030	-,219**	-,109**	,403**	-,414**	-,184**	0,018	1
	Sign.	0,000	0,000	0,000	0,000	0,000	0,000	0,000	0,398	0,000	0,002	0,000	0,000	0,000	0,601	
	N	807	807	807	807	807	806	807	807	807	807	807	807	807	807	807

** significance at .01

* significance at.05

INTRO=introspection; MENT ST= mental state; SOC= socialness; MOR= moralness; ECO=economicity; THEOR= theoretical; QUANT= quantity; CNC=concreteness; IMG=imageability; FAM=familiarity; AoA=age of acquisition; VAL=valence; SEM_DIV=semantic diversity.

Table S2: Mean and standard deviation of exclusivity total scores and for each level of exclusivity

TYPE OF CONCEPTS	**EXCLUSIVITY LEVEL**	**EXCLUSIVITY (MEAN)**	**EXCLUSIVITY (SD)**
Theoretical	LOW (N=77)	12,55	2,05
	MEDIUM (N=93)	17,90	1,53
	HIGH (N=102)	24,08	3,51
	TOTAL (N=272)	18,71	5,33
Economic	LOW (N=83)	12,55	2,11
	MEDIUM (N=88)	17,82	1,55
	HIGH (N=62)	22,81	2,02
	TOTAL (n=233)	17,28	4,45

Quantitative	LOW (N=49)	12,40	2,17
	MEDIUM (N=94)	17,98	1,56
	HIGH (N=102)	22,67	1,63
	TOTAL (N=245)	18,81	4,21
Spatial	LOW (N=39)	11,98	2,21
	MEDIUM (N=51)	17,90	1,58
	HIGH (N=45)	22,78	1,97
	TOTAL (N=135)	17,82	4,67
Social	LOW (N=140)	12,95	2,00
	MEDIUM (N=275)	17,74	1,35
	HIGH (N=47)	21,85	1,56
	TOTAL (N=462)	16,71	3,19
Moral	LOW (N=106)	13,17	1,72
	MEDIUM (N=203)	17,51	1,27
	HIGH (N=12)	20,98	0,53
	TOTAL (N=321)	16,21	2,64
Introspective	LOW (N=112)	12,77	1,99
	MEDIUM (N=264)	17,72	1,38
	HIGH (N=41)	21,19	0,57
	TOTAL (N=417)	16,73	3,02

Mental States	LOW (N=119)	12,77	1,88
	MEDIUM (N=262)	17,72	1,38
	HIGH (N=39)	21,24	0,58
	TOTAL (N=420)	16,64	3,03

Table S3. Mean and standard deviation of all psycholinguistic variables for all types of concepts, for each level of exclusivity (low, medium, and high).

TYPE OF CONCEPTS	EXCLUSIVITY	CNC*	IMG*	FAM*	AoA*	VAL*	AROUSAL	SEM DIV*
Theoretical	LOW (N=77)	2,66 (0,82)	2,61 (0,73)	4,33 (1,26)	10,93 (2,5)	5,07 (1,7)	5,43 (1,33)	4,33 (1,16)
	MEDIUM (N=93)	3,22 (1,14)	3,04 (0,99)	4,43 (1,05)	10,75 (2,31)	5,7 (1,6)	5,40 (1,36)	3,68 (1,12)
	HIGH (N=102)	3,61 (1,27)	3,45 (1,33)	4,35 (1,21)	10,84 (3,03)	5,04 (0,87)	6,11 (1,01)	2,73 (1,24)
Economic	LOW (N=83)	2,95 (0,83)	2,90 (0,7)	4,57 (1,17)	10,09 (2,41)	5,39 (2,17)	4,78 (1,51)	4,33 (1,15)
	MEDIUM (N=88)	3,68 (1,04)	3,42 (1,07)	4,50 (1,15)	10,12 (2,42)	5,29 (1,67)	5,27 (1,33)	3,85 (1,12)
	HIGH (N=62)	4,77 (1,12)	4,42 (1,29)	4,95 (1,11)	10,54 (2,95)	4,99 (1,26)	5,67 (0,96)	2,61 (1,07)
Quantitative	LOW (N=49)	3,07 (0,85)	2,95 (0,75)	4,6 (1,21)	9,23 (2,61)	5,13 (2,12)	5,02 (1,33)	4,70 (1,11)
	MEDIUM (N=94)	3,74 (1,1)	3,47 (1,05)	4,46 (1,15)	9,53 (2,52)	4,87 (1,41)	5,66 (1,02)	4,28 (1,17)
	HIGH (N=102)	4,50	4,38	5,12	9,17 (3,13)	4,91	5,77 (1,00)	3,29 (1,33)

		(1,09)	(1,22)	(1,13)		(1,14)		
Spatial	LOW (N=39)	3,01 (0,80)	3,25 (0,81)	4,83 (1,03)	8,98 (2,19)	5,13 (1,74)	5,20 (1,35)	5,05 (0,98)
	MEDIUM (N=51)	3,89 (1,20)	3,91 (1,08)	4,64 (0,93)	8,52 (2,04)	4,54 (1,24)	5,88 (0,99)	4,75 (0,83)
	HIGH (N=45)	4,53 (1,03)	4,61 (1,02)	5,26 (1,08)	7,31 (2,47)	4,90 (0,86)	6,04 (1,01)	4,24 (1,26)
Social	LOW (N=140)	2,71 (0,83)	2,88 (0,72)	4,94 (1,03)	9,43 (2,31)	5,04 (2,31)	5,06 (1,78)	4,58 (1,00)
	MEDIUM (N=275)	2,58 (1,08)	3,00 (0,99)	5,17 (0,96)	8,81 (2,42)	5,31 (2,47)	4,72 (1,94)	4,10 (0,89)
	HIGH (N=47)	3,88 (1,66)	4,25 (1,41)	5,37 (1,11)	8,41 (3,04)	4,66 (2,05)	5,06 (1,53)	3,57 (1,08)
Moral	LOW (N=106)	2,67 (0,81)	2,81 (0,77)	4,92 (1,02)	9,54 (2,20)	5,11 (2,37)	5,09 (1,93)	4,43 (1,03)
	MEDIUM (N=203)	2,40 (0,99)	2,86 (0,92)	5,13 (0,95)	8,84 (2,34)	5,70 (2,46)	4,54 (1,97)	4,01 (0,82)
	HIGH (N=12)	3,20 (1,34)	3,88 (1,37)	5,39 (1,28)	7,71 (3,19)	5,73 (2,69)	4,16 (1,94)	3,68 (0,96)
Introspective	LOW (N=112)	2,41 (0,64)	2,69 (0,66)	5,08 (1,07)	9,26 (2,38)	4,78 (2,26)	5,28 (1,81)	4,76 (0,88)
	MEDIUM (N=264)	2,21 (0,70)	2,71 (0,78)	5,24 (0,97)	8,76 (2,44)	5,05 (2,49)	4,88 (1,96)	4,22 (0,78)
	HIGH (N=41)	2,33 (0,97)	3,15 (0,92)	5,23 (0,96)	8,58 (2,53)	5,49 (2,54)	4,55 (2,17)	3,93 (0,83)

Mental States	LOW (N=119)	2,43 (0,70)	2,72 (0,74)	5,09 (1,00)	9,26 (2,34)	4,76 (2,24)	5,30 (1,81)	4,75 (0,90)
	MEDIUM (N=262)	2,18 (0,66)	2,70 (0,77)	5,22 (0,99)	8,81 (2,43)	5,09 (2,50)	4,85 (1,97)	4,21 (0,76)
	HIGH (N=39)	2,09 (0,66)	2,90 (0,72)	5,06 (1,08)	9,05 (2,68)	5,55 (2,53)	4,50 (2,17)	3,84 (0,79)

*CNC=concreteness; IMG=imageability; FAM=familiarity; AoA=age of acquisition; VAL=valence; SEM_DIV=semantic diversity.

Table S4: mean and standard deviation of all semantic dimensions for all types of concepts, for each level of exclusivity (low, medium, and high)

TYPE OF CONCEPTS	EXCLUSIVITY	INTRO	MENTAL ST	SOC	MOR	ECO	THEOR	SPACE	QUANT
Theoretical	LOW (N=77)	3,49 (1,18)	3,52 (1,17)	4,07 (1,36)	3,57 (1,29)	3,47 (1,28)	4,24 (0,54)	2,54 (1,25)	2,99 (1,22)
	MEDIUM (N=93)	2,78 (1,59)	2,85 (1,6)	3,37 (1,44)	2,94 (1,47)	3,91 (2,17)	4,33 (0,71)	2,41 (1,43)	3,51 (1,73)
	HIGH (N=102)	1,71 (0,92)	1,78 (0,97)	2,01 (0,85)	1,53 (0,6)	3,29 (2,43)	4,92 (0,93)	2,49 (1,64)	3,97 (2,02)
Economic	LOW (N=83)	3,47 (1,22)	3,48 (1,34)	4,31 (1,2)	3,84 (1,15)	4,63 (0,78)	3,40 (0,85)	2,41 (1,14)	3,46 (1,28)
	MEDIUM (N=88)	2,16 (0,87)	2,17 (0,87)	3,31 (1,32)	2,79 (1,28)	5,59 (1,07)	3,69 (0,88)	2,43 (1,26)	4,47 (1,50)
	HIGH (N=62)	1,32 (0,32)	1,31 (0,41)	2,68 (0,98)	1,77 (0,51)	6,50 (0,64)	3,67 (0,96)	1,91 (0,72)	5,11 (1,16)
Quantitative	LOW (N=49)	3,23 (1,09)	3,11 (1,07)	3,94 (1,29)	3,28 (1,13)	4,34 (1,23)	3,41 (0,82)	3,01 (1,54)	4,72 (0,87)
	MEDIUM (N=94)	2,20	2,14 (0,76)	3,09	2,19	4,58	3,57	3,49	5,22 (0,84)

		(0,81)		(1,44)	(0,85)	(1,97)	(0,96)	(1,68)	
	HIGH (N=102)	1,50 (0,45)	1,53 (0,56)	2,48 (1,29)	1,59 (0,54)	4,51 (2,33)	3,65 (1,05)	3,12 (1,90)	5,54 (0,94)
Spatial	LOW (N=39)	3,51 (1,22)	3,65 (1,19)	3,69 (1,23)	2,65 (0,78)	3,12 (1,08)	3,28 (0,73)	4,57 (0,87)	3,58 (1,58)
	MEDIUM (N=51)	2,31 (1,04)	2,26 (0,90)	3,12 (1,67)	1,86 (0,67)	2,88 (1,41)	3,26 (0,99)	5,03 (1,08)	4,79 (1,35)
	HIGH (N=45)	1,72 (0,66)	1,75 (0,67)	2,03 (1,26)	1,29 (0,36)	2,29 (1,21)	3,32 (1,08)	5,40 (1,21)	5,18 (1,61)
Social	LOW (N=140)	4,10 (1,29)	4,15 (2,29)	4,99 (0,91)	4,13 (1,18)	3,34 (1,29)	3,29 (0,80)	2,44 (1,08)	2,76 (1,13)
	MEDIUM (N=275)	4,88 (1,62)	4,85 (1,59)	4,88 (0,90)	4,20 (1,34)	2,31 (1,50)	2,82 (0,79)	1,68 (0,96)	2,12 (1,12)
	HIGH (N=47)	3,30 (2,12)	3,24 (2,22)	5,00 (1,15)	2,65 (1.03)	2,92 (2,31)	2,58 (0,96)	1,95 (1,13)	2,83 (1,73)
Moral	LOW (N=106)	4,13 (1,26)	4,15 (1,28)	4,96 (1,09)	4,73 (0,77)	3,40 (1,25)	3,28 (0,79)	2,04 (0,70)	2,59 (1,01)
	MEDIUM (N=203)	5,11 (1,47)	5,10 (1,41)	4,74 (1.03)	4,90 (0,90)	2,22 (1,31)	2,82 (0,76)	1,38 (0,38)	1,86 (0,80)
	HIGH (N=12)	4,64 (1,51)	4,56 (2,04)	5,17 (1,49)	4,30 (0,77)	1,68 (0,60)	2,32 (0,55)	1,44 (0,47)	1,40 (0,36)
Introspective	LOW (N=112)	4,79 (0,80)	4,69 (0,97)	4,63 (1,25)	4,01 (1,26)	2,97 (1,15)	3,22 (0,75)	2,39 (1,06	2,50 (0,94)
	MEDIUM (N=264)	5,58 (0,87)	5,51 (0,98)	4,46 (1,18)	4,14 (1,32)	1,81 (0,73)	2,74 (0,72)	1,58 (0,88)	1,88 (0,73)

	HIGH (N=41)	5,68 (1,01)	5,69 (1,37)	3,79 (1,41)	2,97 (0,70)	1,37 (0,33)	2,56 (0,89)	1,63 (1,12)	1,61 (0,51)

Mental States	LOW (N=119)	4,61 (0,99)	4,75 (0,85)	4,55 (1,26)	4,01 (1,25)	2,99 (1,19)	3,25 (0,78)	2,42 (1,05)	2,53 (1,02)
	MEDIUM (N=262)	5,55 (0,96)	5,57 (0,87)	4,46 (1,16)	4,15 (1,32)	1,82 (0,79)	2,76 (0,75)	1,55 (0,81)	1,87 (0,69)
	HIGH (N=39)	5,68 (1,15)	5,91 (1,05)	3,61 (1,20)	2,90 (0,67)	1,39 (0,35)	2,76 (1,14)	1,50 (0,98)	1,64 (0,78)

*INTRO=Introspection; MENTAL ST= Mental States; SOC=Socialness; MOR= Moralness; ECO=Economicity; THEOR= Theoreticiy; QUANT = Quantity.
